# Thermal Welding by the Third Phase Between Polymers: A Review for Ultrasonic Weld Technology Developments

**DOI:** 10.3390/polym12040759

**Published:** 2020-03-31

**Authors:** Jianhui Qiu, Guohong Zhang, Eiichi Sakai, Wendi Liu, Limin Zang

**Affiliations:** 1 Department of Mechanical Engineering, Faculty of Systems Science and Technology, Akita Prefectural University, Akita 015-0055, Japan; zgh131523@163.com (G.Z.); e_sakai@akita-pu.ac.jp (E.S.); 2 College of Transportation and Civil Engineering, Fujian Agriculture and Forestry University, Fuzhou 350108, China; wdliu9054@163.com; 3 MOE Key Laboratory of New Processing Technology for Nonferrous Metal & Materials, College of Materials Science and Engineering, Guilin University of Technology, Guilin 541004, China; zanglimin0705@163.com

**Keywords:** thermal welding by the third phase (TWTP), ultrasonic welding (USW), welding between dissimilar materials, thermoplastic welding technology

## Abstract

Ultrasonic welding (USW) is a promising method for the welds between dissimilar materials. Ultrasonic thermal welding by the third phase (TWTP) method was proposed in combination with the formation of a third phase, which was confirmed as an effective technology for polymer welding between the two dissimilar materials compared with the traditional USW. This review focused on the advances of applying the ultrasonic TWTP for thermoplastic materials. The research development on the ultrasonic TWTP of polycarbonate (PC) and polymethyl methacrylate (PMMA), polylactic acid (PLA) and polyformaldehyde (POM), and PLA and PMMA are summarized according to the preparation of the third phase, welded strength, morphologies of rupture surfaces, thermal stability, and others. The review aimed at providing guidance for using ultrasonic TWTP in polymers and a basic understanding of the welding mechanism, i.e., interdiffusion and molecular motion mechanisms between the phases.

## 1. Introduction

Thermoplastic materials have drawn much attention as alternatives to thermoset materials in many fields due to their advantages such as low cost and effective manufacturing [1]. After being damaged, thermoplastics and their composites can join together based hot melt adhesives in the weld lines under pressure, without the degradation of material properties [2]. USW can overcome the problems such as stress concentrations and delamination due to hole drilling in mechanical fastening, and toxicity, surface modification, and environmental damage in adhesive bonding. Moreover, it can solve the main point relating to the cost of productions. Additionally, there are some techniques that are considered for welding thermoplastics and their composites, i.e., resistance, induction, ultrasonic welding [3], laser welding, and others.

Ultrasonic molding has emerged as a promising replication technology for low and medium volume production of micro-scale parts recently [4]. During a relatively long-term application, it can produce a wide variety of polymeric materials without any thermal decomposition [5] and with significantly reduced energy consumption [6]. Furthermore, metal products that are employed in aerospace, aircrafts, automotive, and microelectronics industries are increasingly replaced by thermoplastic products [7,8,9,10,11,12]. The use of polymer-based parts in automotive could reduce their weight, improve their performance characteristics, and reduce carbon emissions into the environments [13]. Many adhesives and adhesion technologies [14,15,16,17,18,19] are available due to the diversity of used materials, but the toxicity problem during welding remains. This accelerating trend requires a new reliable technology to weld thermoplastics, especially for welding dissimilar materials [20,21,22,23,24,25,26,27,28,29,30,31,32,33,34,35,36,37,38] in industry.

### 1.1. Techniques for Welding Thermoplastics

Thermoplastics are employed in a wide range of applications due to their reduced weight, superior performance characteristics, relaxing stress concentration, and reprocessibility. Usually, it is necessary to use a welding technique for the preparation of structurally complex products, although polymer has excellent processability, and thus a high degree of free shape [39].

According to the physical principles of joining, joints in plastics can be classified as follows: one may distinguish betweemn joints held together by force, shape, or material. The latter category (owing to the rapid development of welding technologies) is used more frequently in several industrial applications. Welding technology is not only necessary for the production, but also for the repair and recycling. In order to prolong the service life of the materials and joints, the waste materials should be impacted and discussed again. Obviously, the key problems are whether the products manufactured or repaired with this technology retain the original properties of the raw materials and how it is possible to optimize the quality of the welded joint [40,41]. Using welding to achieve joints, it is important to produce the strongest cohesion-based joints. The necessary preconditions of welding reveal that a rheological adequate state is necessary to achieve a good quality weld [42]. Joining thermoplastic technologies can be divided into three methods, namely [43]: mechanical fastening, adhesive bonding, and thermal welding.

Mechanical fastening methods include clipping, clamping, screwing, and riveting. Self-piercing rivets and clinch joints have been identified as two types of fasteners with considerably potential use in automotive bodies [44]. Work pieces (WPs) are mechanically fastened together at room temperature in each process. There may be no requirement for the predrilling of holes in the WPs under both cases. The advantages and disadvantages of mechanical fastening are summarized in Table 1 [45,46].

Adhesive bonding is another way for widely used, which is possible to join dissimilar and incompatible materials [47,48]. It has been used car manufacturers in concept cars and low volume niche products [49,50,51,52], e.g., Ford’s AIV, Rover’s ECV3, a limited extent in Honda’s NSX [53] and Mitsubishi. However, no matter how high-performance adhesives such as epoxy or solvent-based adhesive systems are, considerable environmental concerns are remaining. The health and safety hazards require significant costs to provide adequate recycling. Given current environmental concerns, there is the possibility that these substances may be banned in the future. Nevertheless, using polymeric panels to replace the metal ones as an integral part of the injection molding process might not be the best way [54,55,56,57]. Furthermore, releasing harmful byproducts is also an important problem for adhesive bonding of thermoplastics [58]. The in vivo degradation of medical adhesives has not been clearly understood, and degradation byproducts such as formaldehyde have been shown to be potentially toxic to cells [59]. The advantages and disadvantages of adhesive bonding are shown in Table 2 [60,61].

### 1.2. Thermal Welding Methods

Thermal welding methods have been defined as the permanent joining of two materials without the use of adhesive or other chemical products at the interface or weld lines [43]. These methods can be applied to many thermoplastics, and some of them have been established methods used in aircraft, automotive, electron device, packaging, and medical fields [62]. Thermal welding methods commonly mention the application of localized heats, times, and pressures. The eventual aim of thermal welding between polymers is the creation of a seamless joint that can own the same mechanical properties with the matrix materials. There are some methods reported to weld between thermoplastics, which are all sharing the same feature of heat generation at the welded interfaces. There are some ways for thermal welding to be employed for the products. Hot-plate welding, laser welding, friction welding, and other methods have been studied by many researchers for developing the achievable welded strength.

In hot-plate welding, two WPs are set between two heated plates and the weld is expected to achieve according to the melting and solidifying process. Shim [63] used hot-plate welding to weld acrylonitrile butadiene styrene (ABS) polymers by a lap-joint method. They found that welded strength enhanced with the increased plate temperature and contact time. Welded strength was controlled well by the sufficient flow of the molten polymer, while the maximum welded strength of 11 MPa was obtained under 260 °C. Watson [64] welded polypropylene (PP), polystyrene (PS), and poly (phenylene oxide) (PPO) circular tubing, which confirmed that the plate temperature and contact time are the important factors to obtain the maximum welded strength. The strength could reach one of the matrices once selecting suitable welding conditions. Nonhof [65] welded ABS and PP using hot-plate welding, presenting that it was difficult to determine a single optimal welding parameter around mass of productions. They also reported that a system of factorial experimental design was the best way for hot-plate welding, and a shortcoming of the melt residue adhering to the hot-plate surface was emphasized, especially for the thermoplastics with a high melting point [66,67].

In laser welding, a laser beam is employed for irradiation to give energy at the interfaces of WPs, where the thermoplastics can be welded after melting and solidifying. Both butt-joint and lap-joint ways are used, while the key points for the welding effects are laser power, scan speed, irradiation times and absorption properties of the matrices [68]. Laser welding is a high-speed process and can achieve weld instantaneously. So, the effect of heat on the zones around the weld lines is relatively smaller when compared with other welding methods [69]. Potente [70] welded polyetheretherketone (PEEK) by pigmenting carbon black into the WP to help absorb laser energy at the interfaces. Acherjee [71] conducted a transmission laser welding for PMMA and investigated the welded strength and the widths of the weld lines widths. With the enhancement of laser power, welded strength and weld line widths increased when compared with the decrease of the strength and widths as the increased scan speed. Georgiev [72] also employed the transmission laser welding to weld Teflon fluorinated ethylene propylene (FEP) to titanium foil and the interfacial formation was investigated. Amanat [14] reported that, although it cost too much, laser welding was the most suitable technology to weld implantable medical devices due to its instantaneous joint, partial heating, and minimal effect on others.

Friction welding is employed utilizing the heat that is generated by rubbing two welding surfaces of WPs together to melt and join them. Like other thermal welding methods, WPs weld can be achieved under welding pressure after the interfaces cool and consolidate [14]. The most common friction welding techniques are vibration, spin, stir, and ultrasonic welding. For the vibration method, one of the WPs is actionless and the other can vibrate along with the welding surface at the desired frequency and amplitude. The heat generated from vibration can melt the surfaces of WPs and then join them together after consolidation. This technique is limited to the parts with flat mating surfaces, and is mostly applied to weld large size parts, such as intake manifolds and bumper assemblies in cars [69,73]. Also, it requires specialized fixtures, and the thermoplastic to be welded must be stiff enough to avoid deformation during the welding process. Stokes [74,75,76] welded PC, polyethylene terephthalate (PBT), and polyetherimide (PEI) to themselves, where the maximum welded strength could achieve the tensile strength of the matrices. Welded strength of ABS could reach 90–95% of the tensile strength of the matrices, and the damage mechanism was quite different from PC and PEI [77]. Cakmak [78] gave a report related to the amorphous and semi-crystalline PEN, while the welded strength of the crystalline polyethylene 2,6-naphthalene dicarboxylate (PEN) was two times that of amorphous PEN. Moreover, Chung [79] studied the microstructure of the heat-affected zone during the vibration welding process for polyamide butt joints. The heat-affected zone was divided into two distinct areas. One was a central layer which was recrystallized from the molten polymer, and the other was a deformed outer layer that was the result of polymer deformation. The central layer had a higher molecular orientation than the outer layer, and in tensile tests, failure occurred through the interface between the inner and outer heat-affected zone layers. 

### 1.3. Recent Work for Welding Thermoplastics

For polymer welding methods, the conventional external heating technology including hot plate welding [80,81,82,83], ultrasonic [84,85], and vibration [86,87] welding has become the most important ones in industry, relative to the internal or mechanical heating methods. Induction [88,89] and laser [90,91,92] welding fall into the category of radiation/electromagnetic technologies, which set new targets in the plastics welding technology. In the last decades, efforts have been made to improve the present processes and to develop new polymer joining techniques [93].

Czigány [94] welded PP and studied the effects of welding conditions on the welded strength by using a hot-gas welding method. They designed a series of experiments with automatic welding station for the welding results. Solvent [95] investigated the welding in the fabrication of micro fluidic devices for thermoplastics, specifically those made of PMMA. It was found that many high-performance polymers were resistant to solvents, and required the use of specialized solvents if they were available. Amancio [10] evaluated the feasibility of the new technology friction spot welding of thermoplastic using PMMA plate. The new method was that WPs were first fixed in the welding machine and then the sleeve and pin began to rotate in the same direction. Then, the sleeve was forced against the upper joining partner generating frictional heat. Potente [96] studied the laser transmission welding of thermoplastics due to its advantageous properties and the increasing interest in this technology. Polycaprolactam 6 (PA6) and the quasi-simultaneous laser welding process were used, and a model was introduced using the finite elements method to describe melt displacement and temperature profiles for laser through-welding. Grewell [97] investigated the orbital vibration welding and linear vibration welding methods with employing WPs made of PP, PP/polyethylene (PE) copolymer, PC, ABS, and PA. Standard full-factorial designs with each process were carried out and the differences and benefits between the two methods were reported.

Moreover, permeating welding technology depended on infrared such as semiconductor laser, and carbon dioxide laser have attracted much attention in both academic and industrial fields in the latest years [98,99,100,101,102,103,104]. Also, double color molding and sandwich welding methods have been proposed with for the plastic products with high-performance and functionalities due to its simply technology and low cost. Naruse [105] carried out ultrasonic plastic welding using a surface acoustic wave device. There are some advantages to using higher frequencies, namely it makes the joining time shorter, damages of joined parts can be avoided, and positioning accuracy become higher. In their study, PE films were used as WPs and the peak welded strength could reach the tensile strength of the matrix polymers.

## 2. Ultrasonic Weld for Polymers

### 2.1. Ultrasonic Weld Methods

**Figure 1:**
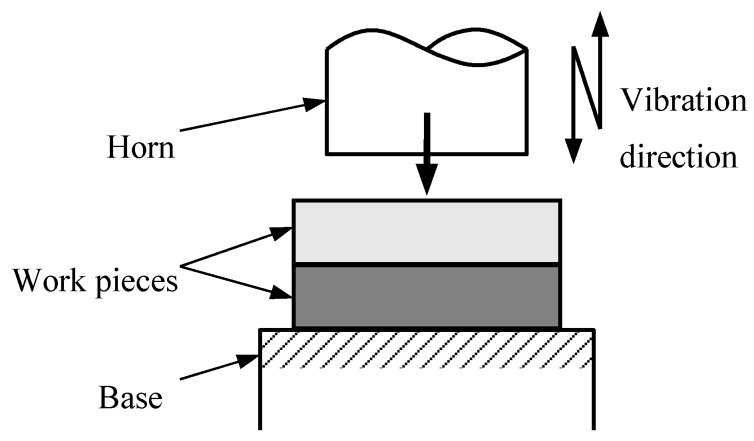
Schematic illustration of USW method. Adapted from Reference [106].

USW for polymer (Figure 1), which uses vibratory energy at high frequencies (20–40 kHz) that are beyond the range of human hearing to produce low amplitude (20–30 µm) mechanical vibrations, is a rapid joint method at the welding interface due to the welding pressure [106]. USW for thermoplastics is a joining technique without additional substances, such as adhesives or solvents. USW possesses well-defined local heating of only the joint area, short cycle times in the range of just a few seconds, and comparatively low cost-efficiency [107]. During the ultrasonic consolidation process, energy generated from an ultrasonic transducer is transferred to a WP in the form of ultrasonic oscillation [108].

There are two different USW types, i.e., near field and far field for the traditional ways [109]. In the near field type, the horn is set close to WPs (up to 6 mm), while in the far field one, the horn is set at distances starting from 6 mm [110]. For welding thick parts of low stiffness or crystalline polymers, the near field type is suitable, while for thin parts of high stiffness or amorphous polymers, the far field type is employed only. 

Commonly, it is necessary to provide enough energy to melt crystals and initiate intermolecular diffusion for the WPs prepared from semi-crystalline polymers such as high-density PE (HDPE), PP, and PE [44]. They are also not suitable for the far-field type because of damping in the welding process. Generally, the stiffness of WPs can improve ultrasonic vibration transmission and enhance the weld ability and properties for USW. Joint design, part geometry, energy requirements, amplitude, and clamping geometry can influence the welded strength and performance. The major welding parameters are welding time, welding pressure, vibration amplitude and holding time, where welding time, welding pressur, and vibration amplitude are the controlling parameters [109].

USW is applied in domestic appliances and medical industry for manufacturing non-implantable medical devices, where hermetic seals and contamination-free joints are required. By using USW, Liu [111] welded amorphous PS and semi-crystalline PP, which indicated that the welding time and amplitude of vibration were important for the welded strength. The welded strength of semi-crystalline PP was 26 MPa, which was three times higher than that of amorphous PS with only 8 MPa. Certainly, for the maximum strength, more energy was needed to achieve weld for PP compared with PS. Chuah [112] investigated three kinds of energy director shapes in the far field type of USW, which are semi-circular shape, rectangular shape and triangular shape. Amorphous ABS and semi-crystalline polyester were welded and the efficiency was calculated as welded strength divided by parent material strength. They emphasized that the welded properties were significantly affected by the shape of the energy director, in which semi-circular shape was confirmed as higher up to 10% weld efficiency than others. In automotive, instrument panels were joined with metallic pieces using metallic inserts [113]. The fabrication of door fascia, bumpers, isolation pieces in the motor housing was current examples of parts welded by ultrasonic energy [114].

**Figure 2:**
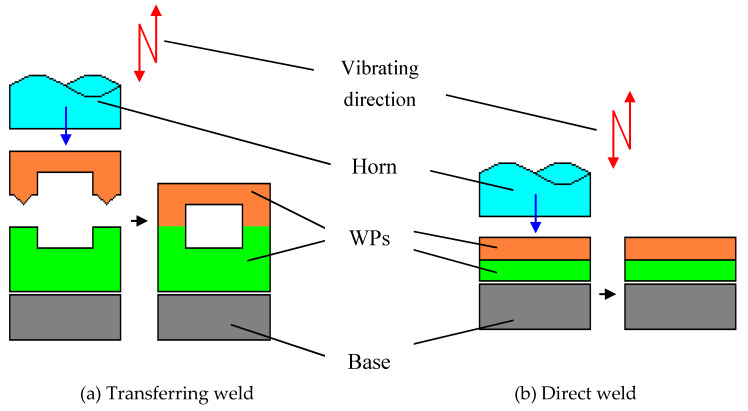
Conventional USW methods. Adapted from Reference [99].

Figure 2 shows the conventional USW method for polymers. The horn fixes to the ultrasonic transducer works on one of the WPs. That is to say, the horn is vibrating out of the weld line and the ultrasonic energy needs to be transmitted by one of WPs, which are always used between identical polymers [115].

Advantages and disadvantages of USW are shown in Table 3. Other major problems, as observed in vibration welding, include the damage of electronic components [112]. 

Therefore, the most troublesome problem is that ultrasonic weld is difficult to weld dissimilar materials. Moreover, once the size and shape of the WPs are changed, the horn may not remain the previous vibration so that the weld cannot be achieved normally.

### 2.2. Weld Between Dissimilar Polymers

It was reported [116] in 2007 that weld for dissimilar polymers was the necessary and efficient processing technology for the excellent materials showing their functions efficiently or the situation of when a single kind of product needs to be employed in a different environment (temperature, load, etc.). Usually, the welds between dissimilar materials are carried out in these fields: welding related to biology-body and artificial organs of nano-biomedicine, electronics technique, energy technology in generating and storing electricity, automotive field with welding between soft and hard materials (organic-matters and metals, insulators), aerospace field with welding between functional materials (semiconductors and metals, insulators), and so on. That is to say, the welding technique for dissimilar materials will be very important and essential in many industrial fields today.

Mechanical fastening and adhesive bonding methods are the traditional ways to weld dissimilar materials. Filho [117] gave the comparison for the bonding methods, where the field of welding for dissimilar plastics was still a new area in joining technology nowadays in several industrial areas. The development of new materials and fabrication techniques has become a matter of success for industrial sectors such as transportation. Polymers, polymeric composites, and polymer–metal structures are being increasingly employed in several products mainly due to the associated weight savings. Conventionally, an ideal component should not show any disadvantages that would deteriorate mechanical strength after welding. Nevertheless, the sizes of WPs are usually limited by their production process. Therefore, the understanding and development of available and new joining techniques is a key subject in industry. 

For the weld between dissimilar polymers, other techniques such as hybrid joining method and plastic injection over molding in metallic perforated parts are being currently exploited [118,119]. Ageorges [120] reported a review for thermoplastic matrix composites by using fusion-based joining methods. All of the presented welding methods for plastics were almost adequate for thermoplastic composites, in which the resistance welding was the most popular technology for application. But friction welding was not suitable due to the microstructural deterioration of the composite through fiber breaking or realignment. McKnight [121] proposed to weld carbon fiber reinforced polyetherketone composite using resistance welding and the welded performance and weld line structure were analyzed. Ageorges et al. [122] gave a report for welding glass fiber and carbon fiber reinforced polyetherimide composite laminates. Other examples of polymer composites welding can also be found [123,124].

In brief, the welds between dissimilar polymers are almost limited by mechanical fastening and adhesive bonding ways, which bring problems according to their disadvantages. Therefore, it is necessary to develop the novel weld method.

### 2.3. New Ultrasonic Welding Application for Polymers

**Figure 3:**
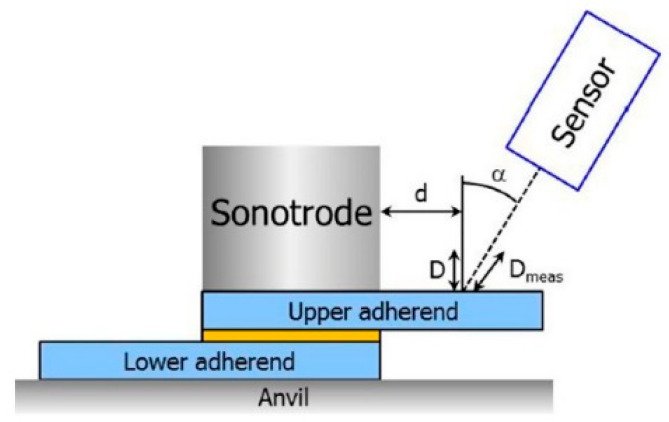
Schematic drawing of determination of amplitude transmission using a tilted sensor. Adapted from Reference [136].

There are considerable research literatures on the USW process for thermoplastic materials at the coupon level [125,126,127,128,129,130,131,132,133,134,135]. The welded condition parameters including welding time, welding pressure, and vibration amplitude, were investigated and the relationship between the welded ability and the parameters were reported. For energy director (ED) USW technique, solidification force and time and shape of EDs were also discussed [136]. Since the heat generation and ultrasonic power could be dissipated during the welding process, it was essential to make sure the ultrasonic parameters such as ultrasonic amplitude, power and vibration time. Palardy [136] proposed an original experimental and modeling approach towards a better understanding of the hammering effect. It consisted of an experimental static welding setup with a high frequency laser sensor and a finite element model as shown in Figure 3. A numerical, multiphysical model based on the framework developed by Levy [137] was employed to evaluate the changed power according to USW process by amplitude transmission measurements. The results showed that it was possible to obtain a good estimation of the vibration transmitted to the upper adherend from laser measurements close to the sonotrode, the effect of which was shown to decrease during the welding process, due to the heating of the interface which directly affected further heat generation. Though these might be potential for evaluating the vibration transmitted of the top WP using the laser sensor, it had been confirmed decrease during the welding process because the heat generated at the interfaces would give effects on the further ultrasonic heat production. Additionally, this might be another way to analyze the welding process, especially for the heat generation, but the welding method was limited and similar to the conventional USW.

**Figure 4:**
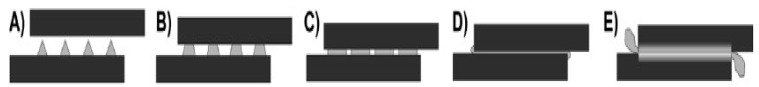
Power and travel curves as function of energy for the initial weld, produced with a travel of 0.5 mm. Adapted from Reference [142]. (**A**) intact EDs; (**B**) collapse of EDs due to melting; (**C**) partial flow of the EDs and melt-front arrest; (**D**) remelting of ED melt-fronts and subsequent flow out of the overlap; (**E**) complete ED squeeze out from the welding interface, with melt and partial flow of the matrix beyond the first adherend layer.

Ultrasonic guided waves (GW) are widely acknowledged to have high potential application [138,139]. Over the last two decades, some studies have focused on finding reliable ways of correlating changes in GW propagation with realistic defects in joints. Singher [140,141] investigated the interaction of ultrasonic GW with adhesively bonded single lap metallic joints produced with different surface treatments. Each surface treatment resulted in different bond strength, while the strength should be enhanced for a higher level. Pedro [142] presented the propagation of ultrasonic GW in ultrasonically welded thermoplastic composite joints in Figure 4. They studied the effects of weld manufacturing defects on GW transmission across the joint and triangular (EDs) integrated into the lower composite adherents enabled the production of defective joints in a controlled manner. They concluded that it was only possible to detect and distinguish the two defective scenarios by combining the characteristic frequency and time analysis at last.

Natesh [143] presented a study undertaken with an objective to establish USW process for joining polymer blends expressed to aid the eco-friendly qualities desired in manufacturing sectors. PC and ABS blends were welded after creating suitable parts with energy directors using injection molding techniques. Finally, only a welded strength of 6.02 N·mm^−2^ was achieved. 

**Figure 5:**
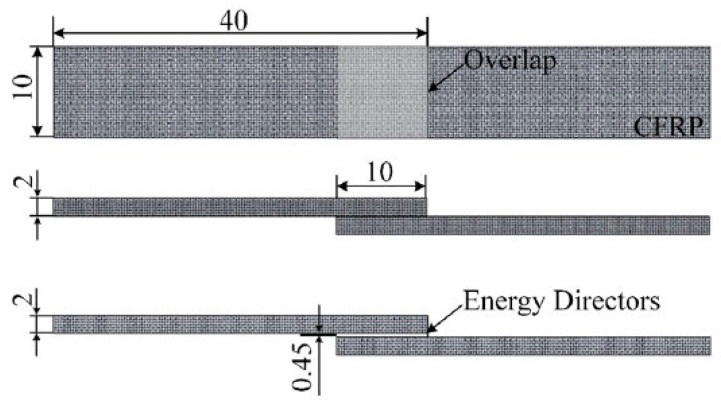
Schematic illustration of joint configuration USW (dimensions in mm). Adapted from Reference [144].

Wang [144] welded carbon fiber/PEEK (CF/PEEK) composites assisted by ultrasonic as shown in Figure 5. They reported the influence of vibration time and ED on the joints, in which the heating rate could be accelerated by using flat ED, and the maximum temperature was lowered. By using flat ED, the lap shear strength of the joint could reach 28 MPa, which might be necessary to be further enhanced by using other ways such as surface modification.

**Figure 6:**
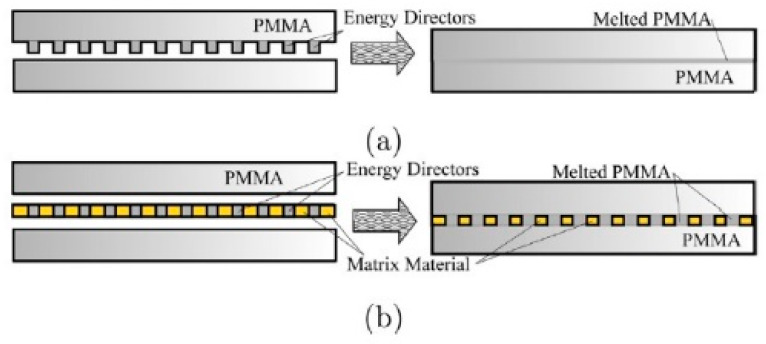
Illustration of polymer using (**a**) conventional and (**b**) composite film USW. Adapted from Reference [145].

Chan [145] presented a composite film of PMMA microspheres in polydimethylsiloxane (PDMS), which was used as the fusion layer to avoid trapped air and to restrict the flow of the melted polymer during welding (Figure 6), compared with conventional one of (EDs) melt and flow across the surface of the samples, resulting in a thinner fusion layer. Although the welded strength in this methodology is limited by the particle size of the PMMA microspheres, it is a way to change the (EDs) into the layer without shape modification. Therefore, the welded strength will be focus of the following work.

In summary, for all of the latest research on the USW method, it is concluded that the WPs have been changing from polymeric materials to their composite (or blends). Moreover, (EDs) are popular and draw much attention by many researchers. The compounds, structure, shapes and sizes are different from each other, though they are all melted and welded into the weld lines. Furthermore, energies coming from ultrasonic vibrations are all sent from one WP to another, which might cause the inhomogeneity of the weld line and residual stress at the interfaces. Finally, welded strength is important point for welding applications in industry, and so it is necessary to be further enhanced.

**Figure 7:**
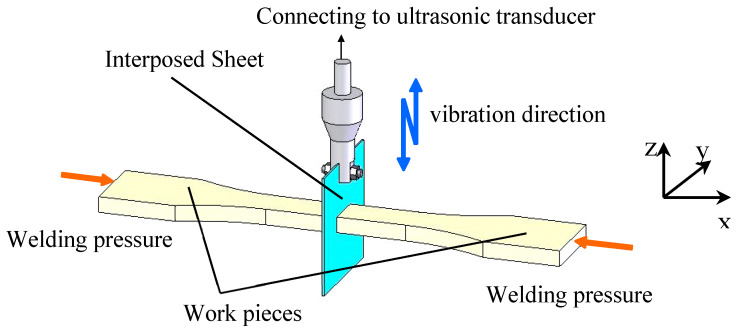
Welding principle schematic drawing followed by ultrasonic TWTP technology. Adapted from Reference [146,147].

Therefore, the ultrasonic TWTP method between polymers has been proposed, which is an effective way to solve the above-mentioned problems and introduced to more widely fields. Schematic drawing of ultrasonic TWTP technology is shown in Figure 7 [146,147]. According to the slide guide (x direction), WPs are installed on the WPs holders, and the third phases of interposed sheets (IPSs) are fixed on the sheet holders (z direction), which are connected to an ultrasonic transducer. Then, IPSs are set between the two WPs and the welding pressures are given by different level (x direction). Abrasive paper is designed (y direction) to modify the welding surface roughness of WPs. At last, after the parameters of ultrasonic vibration are set, the weld can be achieved.

The effects of the technology on welding parameters have been investigated for both identical and dissimilar polymers. The ultrasonic TWTP technology can solve the above problems and avoid the disadvantages of the traditional USW method in Table 3. It may be applied widely in industry, so the following statements are the application of this technology in welding different pairs of polymers.

## 3. Ultrasonic TWTP Technology of PC and PMMA 

### 3.1. IPS Preparation for Ultrasonic TWTP Between PC and PMMA

**Figure 8:**
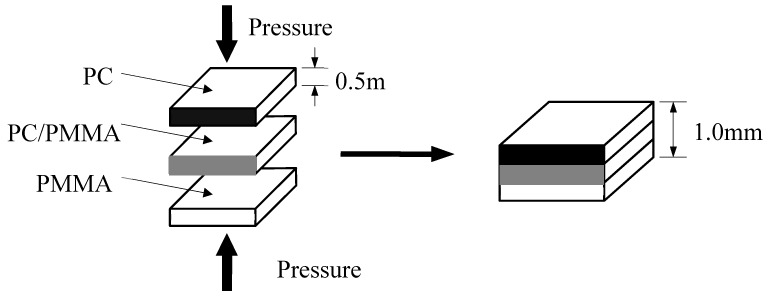
Schematic diagram of IPS preparation for PC-PMMA weld. Adapted from Reference [148].

IPS for ultrasonic TWTP weld between PC and PMMA were prepared as functional gradient materials due to the different physical properties of matrices. Figure 8 illustrated the preparation methods of the IPS [148]. PC/PMMA composites were prepared by injection molding with a volume fraction of PC:PMMA = 1:1. Then, PC, PMMA, and PC/PMMA composites were reshaped to layers with the thickness of 0.5 mm by hot-press, respectively. Finally, the three different layers were used to mold and the IPS was obtained after modifying the thickness to 1 mm.

### 3.2. Mechanical Properties of IPS for Ultrasonic TWTP Between PC and PMMA

**Figure 9:**
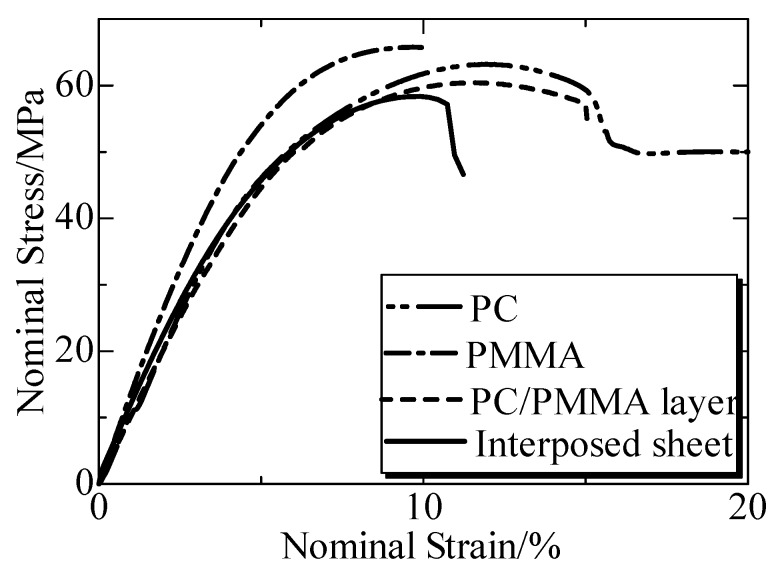
Nominal stress-strain curves of PC, PMMA, PC/PMMA composite and IPS. Adapted from Reference [149].

The mechanical properties were shown in Figure 9 with the nominal stress-strain curves. The tensile strength of the IPS (PC is yield strength) was 60 MPa. The matrix PC showed a necking deformation and the nominal stress weakened sharply when the strain reached yield strength, showing a typical ductile character. PC continued its elongating with the necking deformation, where the stress slowly increased and then fractured finally when the strain was above 110%. In contrast, the elasticity of PMMA was higher than that of PC, but the necking deformation was not observed, presenting a typical brittle character. The IPS made of PC and PMMA showed a brittle character, which was similar to PMMA. It was suggested that IPS was a brittle material with a tensile strength of 58 MPa. Meanwhile, IPS exhibited a good compatible structure, resulting in good compatibility with PC and PMMA due to the absence of the phase separation [149] at the rupture surface. So, the IPS prepared from PC and PMMA was considered to be appropriate as the third phase for welding PC and PMMA.

### 3.3. Welded Strength of Ultrasonic TWTP Between PC and PMMA

**Figure 10:**
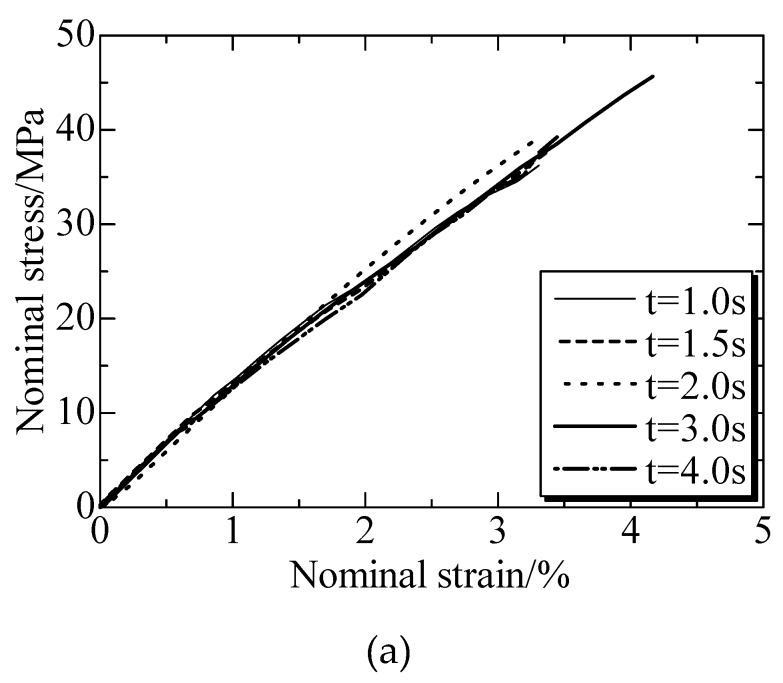

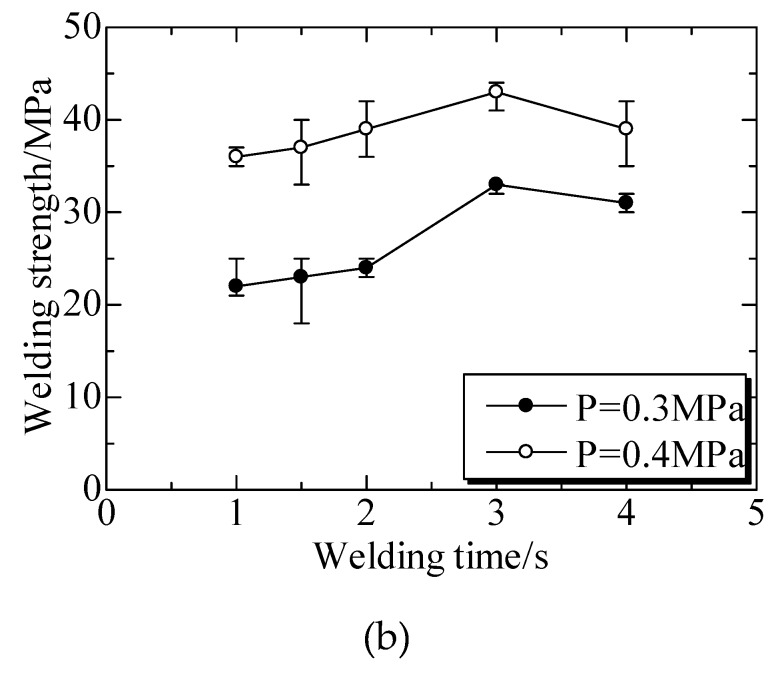
Welded strengths at different welding times. (**a**) Nominal stresses and strain at different welding times under welding pressure of 0.4 MPa; (**b**) Welded strengths at different welding times. Adapted from Reference [149].

The welded strengths at different welding times were shown in Figure 10. The tensile stress-strain curves (Figure 10a) under a welding pressure (*P*) of 0.4 MPa indicated that the tensile stress increased linearly with the strain prolonging, and the samples was broken in the strain of below 4% [149]. This character was same for all the curves in Figure 10a, even though they were welded at different conditions. Figure 10b illustrated the curves of tensile strengths versus welding time (*t*), where the welded strengths presented same trend as welding time under different P. Under welding pressure of 0.3 MPa, welded strengths enhanced with the increase of welding time firstly and then decreased with the time. However, the effect of welding time from 1 to 4 s on the strength were not significant when compared to that of welding pressure. It was concluded that the strength could be enhanced with prolonging time, but the change was limited by presses. 

**Figure 11:**
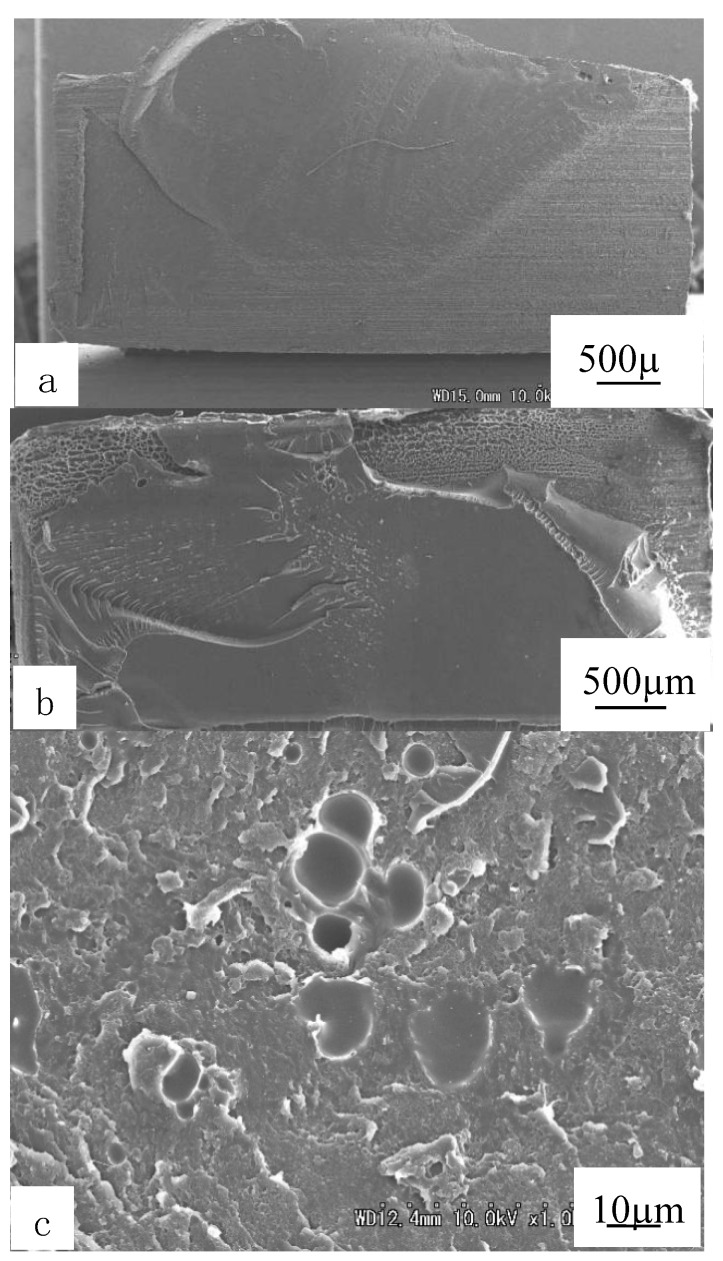
SEM images of ruptured surfaces of welded interfaces of PC side under welding pressure of 0.3MPa. (**a**), (**b**), (**c**) are under welding time of 1.5 s, 3 s and 4 s. Adapted from Reference [149].

The SEM images of ruptured surface were shown in Figure 11. The welded areas were calculated, where the welded strength was enhanced with the increased areas. With the increase of welding time, more frictional heat was produced, welded areas were gradually increased. It could be seen that the strength was weak at 1.5 s and the area was also very low (Figure 11a), and the maximum strength was obtained when the areas were full (Figure 11b). Additionally, the air bubbles at ruptured surfaces existed when welding time was longer than 4 s (Figure 11c). It was considered that the polymers decomposed at the local part and gas was released because of the longer ultrasonic vibrating and heating. The air bubbles might be the reasons for the decreased strength when the welding time was higher than 3 s.

**Figure 12:**
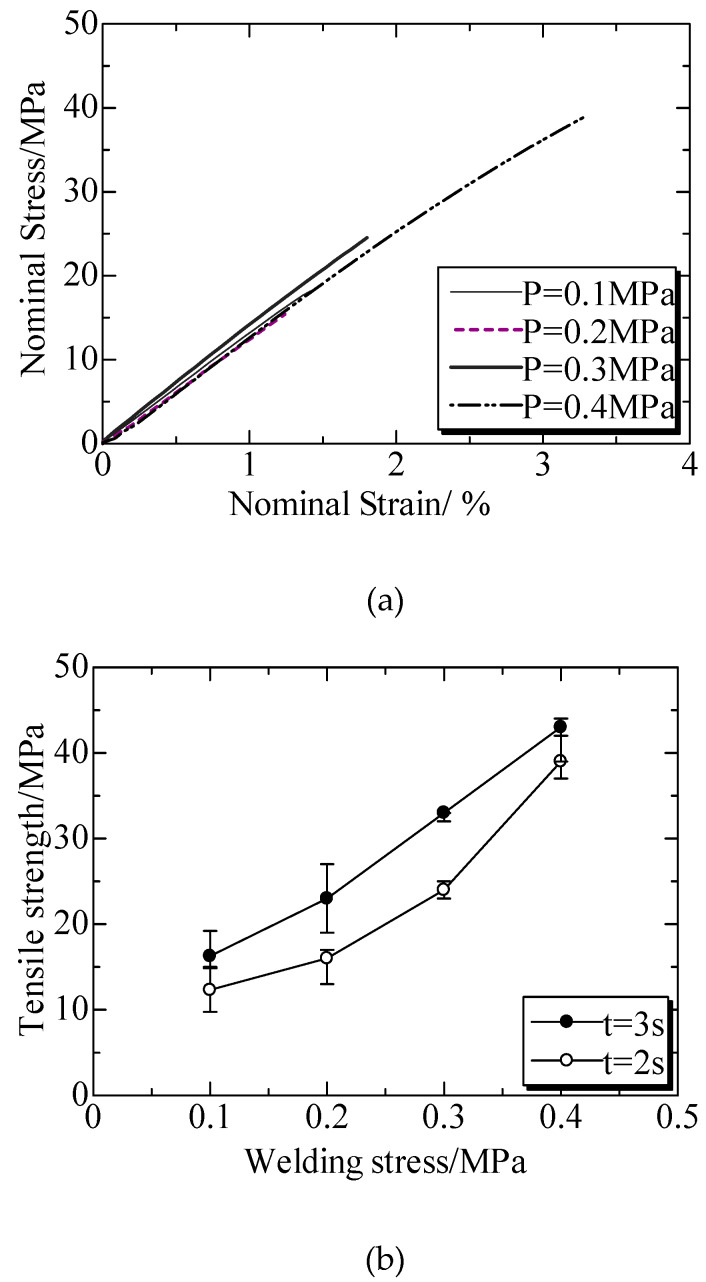
Welded strengths at different welding pressures. (**a**) Nominal stress and strain at different welding pressures under welding time of 2 s; (**b**) Welded strengths at different welding pressures under welding times of 2 s and 3 s. Adapted from Reference [149].

The welded strengths at different welding pressures were shown in Figure 12. Figure 12a illustrated the tensile stress–strain curves at a welding time of 2 s. Compared to that of welding time, the effects of welding pressure on the tensile stress presented a same trend, showing a linear effect and the specimens broken in the strain of below 4% [149]. Figure 12b shows the dependence of tensile strength on welding pressure, and the strength enhanced as the increased welding pressure, which was more significant than those of welding time. However, when the pressures were higher than 0.4 MPa, the ultrasonic plastic welds often displayed errors and cannot weld normally due to the excessive friction force and power [149]. Moreover, excellent welding pressure was not observed due to the fact that the rigidity of the functional gradient IPS was stronger than that of matrix PC. Therefore, IPS could vibrate together with the ultrasonic vibration.

**Figure 13:**
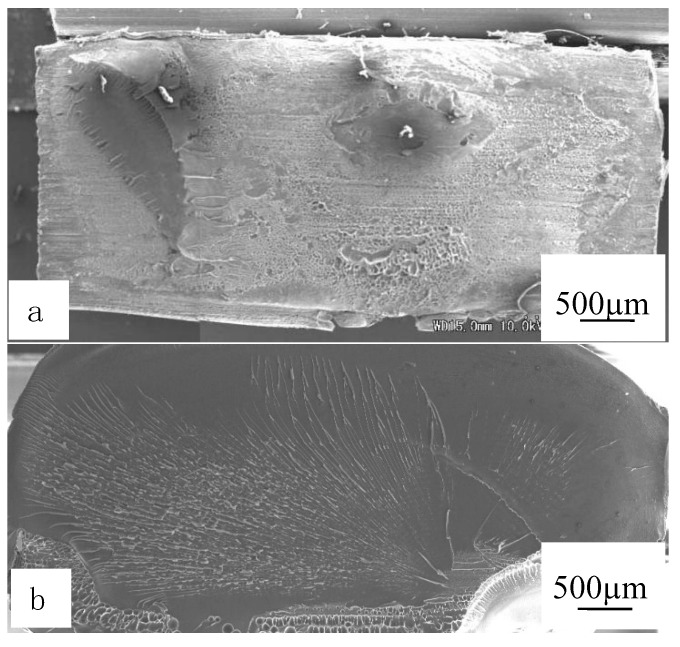
SEM images of rupture surfaces under welding time of 3.0 s. (**a**) and (**b**) are at welding pressures of 0.1 MPa and 0.4 MPa. Adapted from Reference [149].

The SEM photos of ruptured surface at a welding time of 3.0 s were shown in Figure 13. As shown in Figure 13a, when the welding pressure was weak (0.1 MPa), there was little frictional heat produced, welding areas were small, and the strength was weak obviously. According to Figure 13b, more heats at the interface would happen with the improved welding pressure. Therefore, the wider the welding areas were, the stronger the welded strengths. According to the analysis above, the welded areas and the strengths were linearly related to each other. Obviously, the strengths could be supposed by the welded areas and the different strengths could also be investigated by the welded areas of the interfaces.

### 3.4. Interfacial Structures Analysis

**Figure 14:**
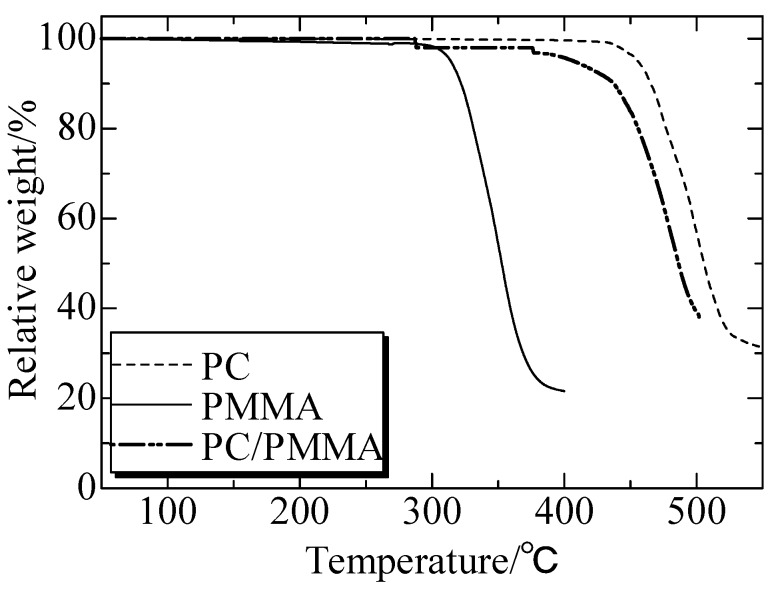
TG test of PC, PMMA and PC/PMMA. Adapted from Reference [149].

Figure 14 illustrated that the decomposition of PC and PMMA started at 450 °C and 300 °C respectively. Air bubbles could be brought easily at the hyperthermal interface between PMMA and IPS, which was proven by the images of the interface.

With the ultrasonic TWTP technology, the welding of dissimilar materials from PC and PMMA was achieved successfully. Based on the analysis for welded strength and interfacial structure, a shorter welding time or weaker welding pressure was inadequate, the frictional heat produced at the interfaces was not enough, welding areas were small, and thus the welds could not be achieved appropriately, and the welded strengths were weak. An excessively long welding time or excessively strong welding pressure is harmful, with much heat produced accompanied by air bubbles at interfaces due to the materials decomposing. This was the reason for the welded strength not reaching the lever of the block materials. Therefore, appropriate welding time and pressure could obtain the optimum value of welded strength.

Therefore, to achieve the maximum welded strength, the increment of welding area and the removal of air bubble were important. Moreover, shorter welding time and higher welding pressure might be effective for ultrasonic TWTP for PC and PMMA. Air bubbles were at the bottom edge although that was the interface of the top strength specimens for PC and PMMA ultrasonic TWTP. It was suggested that the bubbles could be squeezed out of the interfaces with the melting plastics resin together. Finally, welded strength was more sensitive to welding pressure when compared with welding time.

## 4. Ultrasonic TWTP Technology of PLA and POM

### 4.1. Welded Strength for Ultrasonic TWTP Between PLA and POM

**Figure 15:**
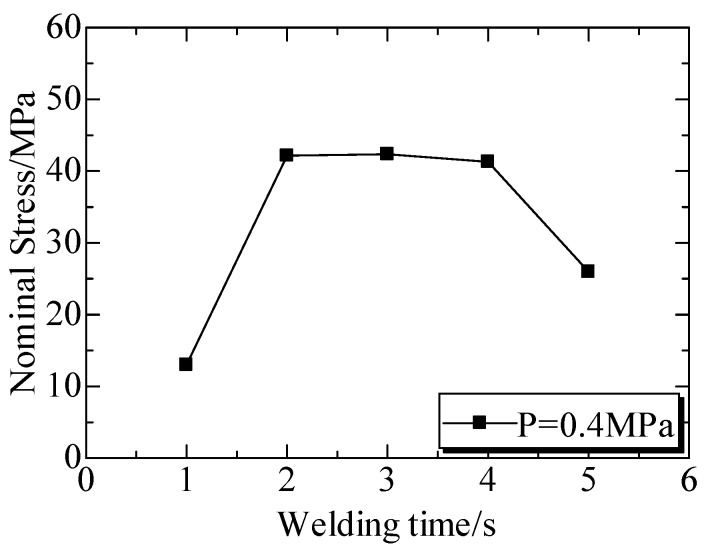
Welded strengths of PLA and POM at different welding times under welding pressure of 0.4 MPa. Adapted from Reference [150].

The ultrasonic TWTP between PLA and POM was achieved because these two matrices were widely employed in industry. The welded strengths of PLA and POM under a welding pressure of 0.4 MPa at different welding time were shown in Figure 15. The welded strengths significantly increased and then decreased with the increase of welding time and the maximum welded strength (43 MPa) was achieved at 2 s and 4 s. It was suggested that the strength could reach the optimum value rapidly under the higher welding pressure and then become weak when the time was too long. It could be considered that ultrasonic TWTP between PLA and POM could be achieved earlier than between PC and PMMA.

**Figure 16:**
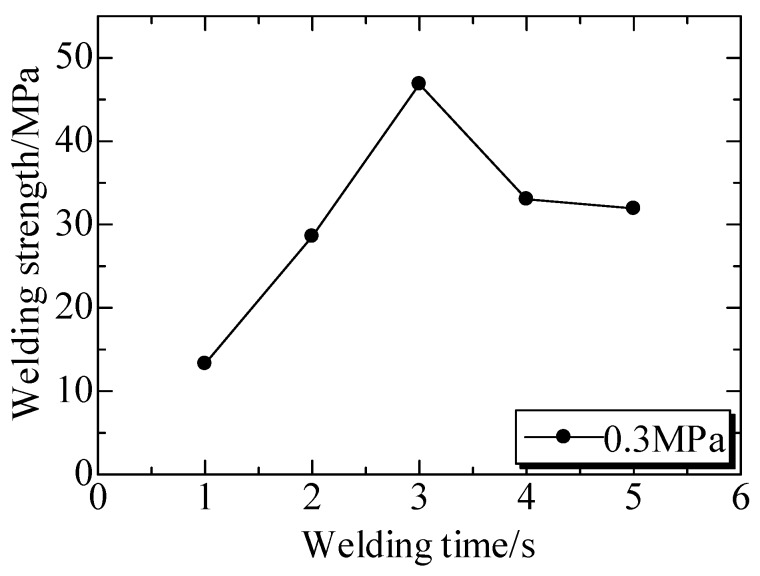
Welded strengths at different welding times under welding pressure of 0.3 MPa. Adapted from Reference [150].

Figure 16 showed the welded strengths at different welding time. It could be observed that welded strengths generally increased firstly and then decreased with the increased welding time and the maximum strength of 47 MPa was achieved at 3 s. This was quite different from the one under 0.4 MPa. The optimum strength was not kept for a long time, and was amazingly higher than the reported results.

### 4.2. Interfacial Structure and Mechanism Analysis

**Figure 17:**
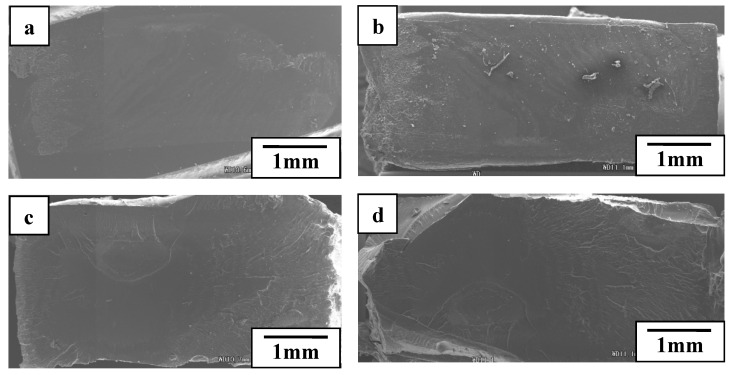
Rupture surfaces of welded interface between POM and PLA under welding pressure of 0.4 MPa. (**a**) is POM side at welding time of 1 s, (**b**) is PLA side at welding time of 1 s, (**c**) is POM side at welding time of 2 s and (**d**) is PLA side at welding time of 2 s. Adapted from Reference [150].

Figure 17 showed the rupture surfaces of welded interface between POM and PLA under a welding pressure of 0.4 MPa and the welding times of 1 s and 2 s, respectively. It was observed that matrices and IPS were melted enough at the welding time of 1 s, and lots of non-weld areas were remained, supposing a weak welded strength. IPS were not melted enough and remained on POM side, which was joined to POM matrix. The IPS surface on PLA side was very tidy compared with the PLA surface, supported by the melted areas as shown in Figure 17b. It was suggested that POM molecules could diffuse into IPS early than the one of PLA side at a short welding time. The melting point of POM was lower than that of PLA. This resulted in molecular chains of POM beginning to diffuse earlier and easier, and thus the weld could be carried partly under the same heat produced. Additionally, IPS was melted completely at the welding time of 2 s, the welded area was full, and a high strength was obtained. 

## 5. Ultrasonic TWTP Technology of PLA and PMMA

### 5.1. IPS Preparation for Ultrasonic TWTP Between PLA and PMMA

PLA/PMMA blends were prepared as IPS phase to weld PMMA and PLA [148]. PLA/PMMA blends with a volume of 1:1 were molded by injection molding and then modified to a thickness of 1 mm via a hot-press.

### 5.2. Mechanical Properties of IPS for Ultrasonic TWTP Between PLA and PMMA

**Figure 18:**
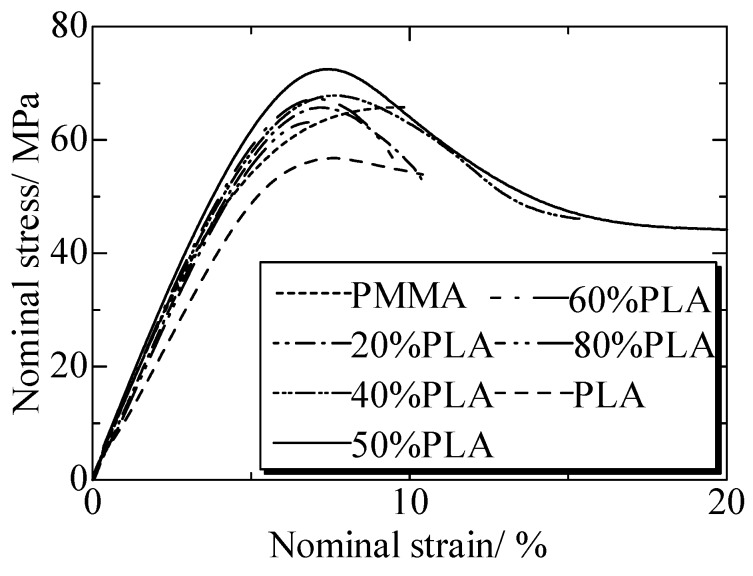
Tensile strengths of matrices and PLA/PMMA blends. Adapted from Reference [148].

**Figure 19:**
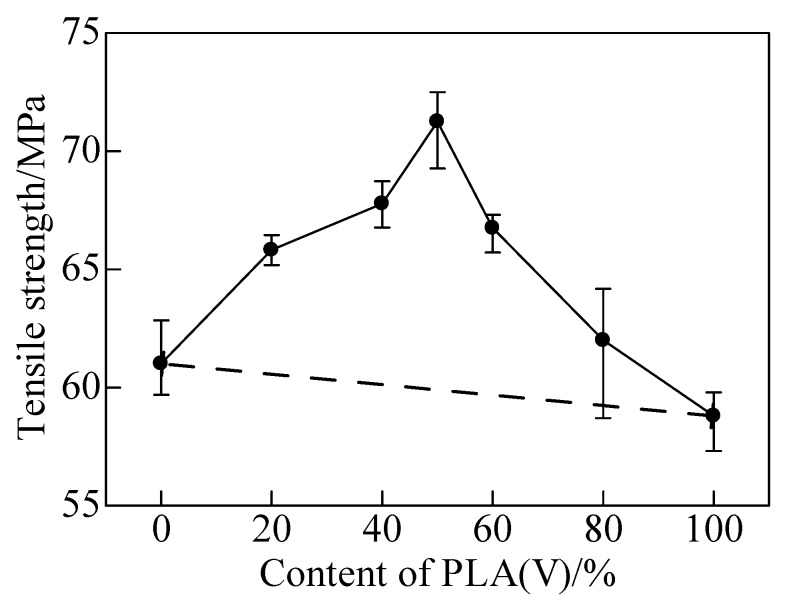
Tensile strengths of matrices and PLA/PMMA blends. Adapted from Reference [148].

Figure 18 gave the tensile strengths of matrices and PLA/PMMA blends. PLA and PMMA were typical brittle polymer with the yield strengths of 58 MPa and 61 MPa, respectively. Their nominal strains were about 10%. With the increased content of PLA, the yield strength of PLA/PMMA blends enhanced (Figure 19).

Dramatically, the yield strength of PLA/PMMA blends with 50% PLA was the highest and the strain was about 180%. According to blend principle, the tensile strengths of blends should be in the range between PLA and PMMA matrices. However, for the PLA/PMMA blends, the tensile strength was enhanced. The maximum yield strength was significantly enhanced to about 73 MPa, which was 18% higher than PLA, showing a typical ductile characteristic. It was indicated that the strength and plasticity of the blends were much enhanced compared with the matrix polymers [148]. The third phase material was the key material of weld for ultrasonic TWTP. High yield strength and plasticity were important and necessary to improve and relax the residual stress inside after weld. For the third materials of ultrasonic TWTP, all PLA/PMMA blends were suitable for employment as IPS. Therefore, the PLA/PMMA (Vol. 50% content of PLA) blend was employed for the following weld of PLA and PMMA due to its optimum properties.

**Figure 20:**
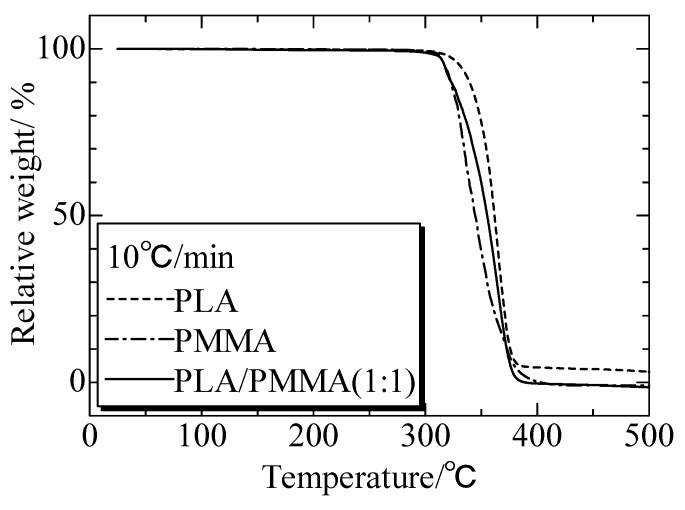
TG analysis of PLA, PMMA, and PLA/PMMA blends of 1:1. Adapted from Reference [151].

Figure 20 presented the TG analysis of PLA, PMMA, and PLA/PMMA blends. All of the matrices and blends showed a same decomposition temperature of 320 °C. The thermal properties of the blends were almost same as the matrix, and thus the third phase IPS would not decompose before the matrices.

### 5.3. Welded Strength of Ultrasonic TWTP Between PLA and PMMA

**Figure 21:**
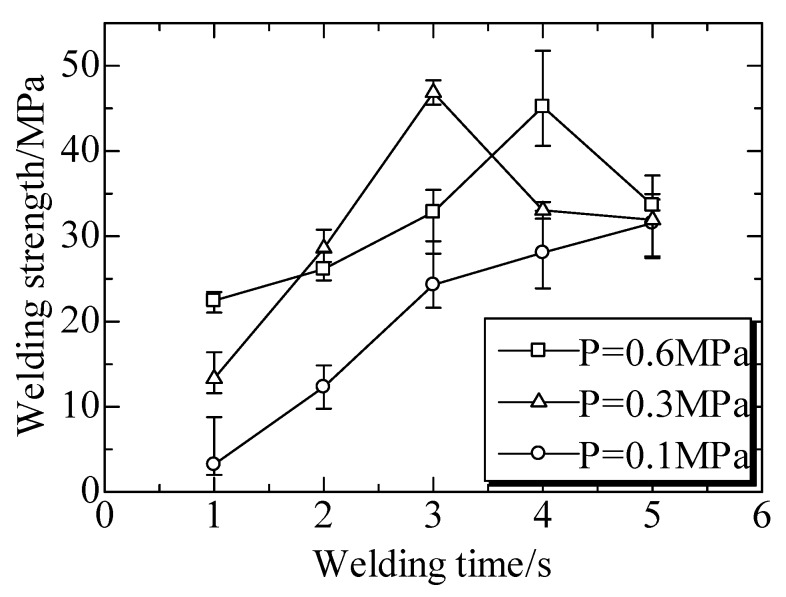
Welded strengths at different welding times under different welding pressures (*P*). Adapted from Reference [151].

The welded strengths at different welding times under different welding pressures (*P*) are shown in Figure 21. The welded strengths generally increased with an increase of welding time from 1 to 3 s under any welded pressure. The maximum welded strengths of 47 MPa and 45 MPa were achieved under the welding pressure of 0.3 MPa and welding time of 3 s, and welding pressure of 0.6 MPa and welding time of 4 s, respectively.

**Figure 22:**
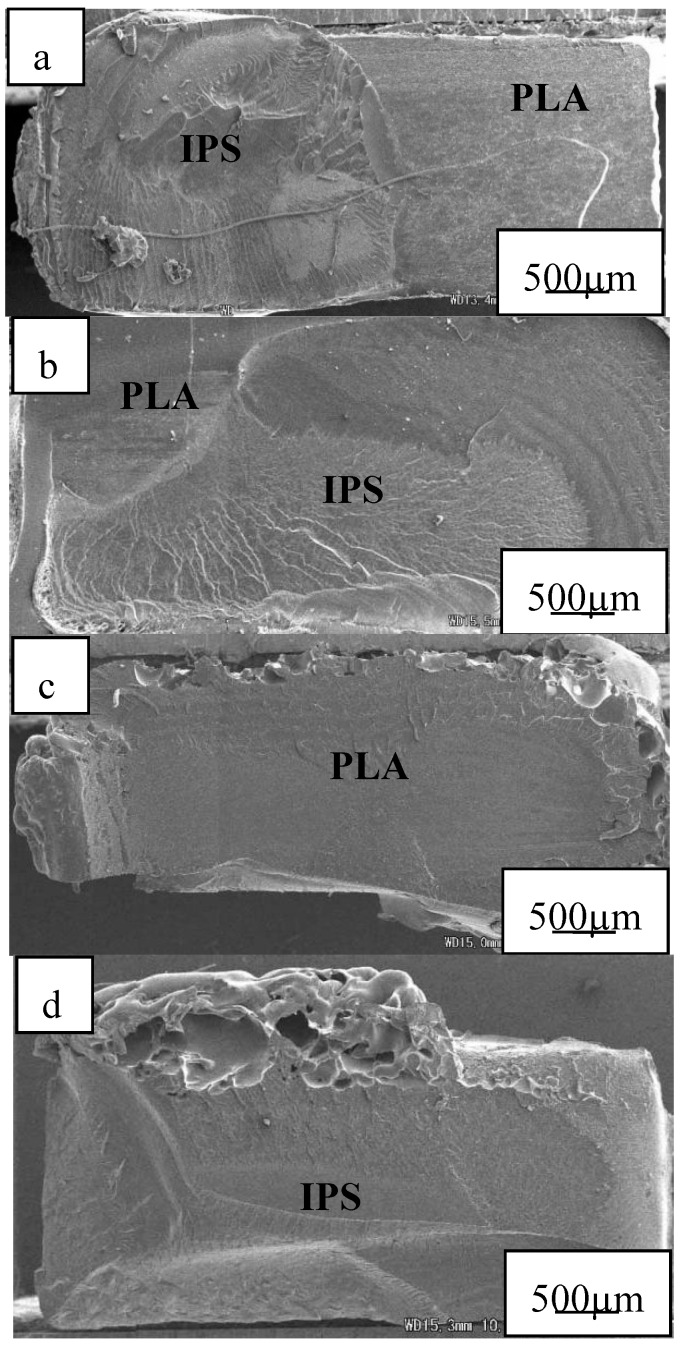
SEM images of rupture surfaces under welding pressure of 0.3 MPa and welding time of (**a**) 1 s, (**b**) 2 s, (**c**) 3 s and (**d**) 5 s. Adapted from Reference [151].

These were similar to the welds between PC and PMMA as well as that between PLA and POM. The rupture surfaces images of welded interface were shown in Figure 22. This indicated the processes of vibrating, heat production, and welded areas enlarging. The decrease of welded strength suggested that too long a welding time or excessively strong welding pressure were harmful due to the interface overheating.

Therefore, ultrasonic TWTP between PC and PMMA, PLA and POM, PLA and PMMA were all achieved using the third phases of IPS prepared from the matrices. Welded strength could be more than 80% of the tensile or yield strength of the matrices. Appropriate welding conditions were necessary for the optimum welded strength, but too long a time and high a pressure would make them weak and induce damage due to the overheating and decomposing.

## 6. Molecular Interdiffusion Analysis of Ultrasonic TWTP Technology

### Interdiffusion Analysis of Ultrasonic TWTP

**Figure 23:**
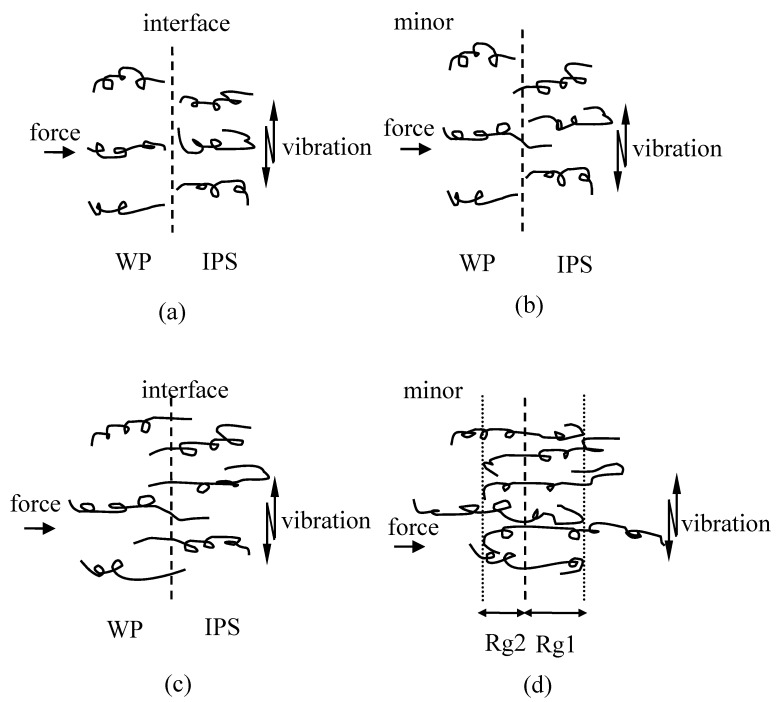
Schematic drawing of macromolecular inter-diffusion of IPS and WPs. Adapted from Reference [147]. Temperatures of the interfaces are (**a**) under room temperature; (**b**) higher than glass transition temperature; (**c**) above melting point; (**d**) back to room temperature.

The schematic drawing of macromolecular inter-diffusion was shown in Figure 23. When the temperature was higher than glass transition temperature (*T*_g_), the molecular chains became loos and began to move due to the frictional heat (Figure 23b). Then, when the temperature reached above the melting point (*T*_m_), the material softened and melted so that molecular chains became free and could inter-diffuse across the weld interface (Figure 23c) [147]. Eventually, the whole chains forgot their initial conformation, and the center of mass diffusion took places, and thus welds could achieve. It could give the welding process of the molecular motion and show the reason for why IPS was partly welded to one matrix side at first. For the most important, they could explain the reason for the successes of weld for dissimilar materials.

## 7. Conclusions and Work in Future

USW is a convenient way for polymers welds, and it is a promising method for welds between dissimilar materials. As a result, the ultrasonic TWTP method has been proposed and confirmed as an effective technology. 

This review focused on the thermal welding methods for polymers. The ultrasonic TWTP technology of PC and PMMA, PLA and POM, and PLA and PMMA were investigated according to the third phase preparation, welded strengths, SEM images of rupture surfaces, thermal stability and others. The welded strength could reach more than 80% of the tensile or yield strengths of the matrices. Materials for the third phases of IPS were suitable for the weld due to their excellent mechanical and thermal properties, where functionally gradient materials were the most important for the most excellent ultrasonic TWTP. Moreover, appropriate welding conditions were necessary for the optimum welded strength, but too long a time and high a pressure could make them weak and induce damage due to the overheating and decomposing.

Finally, it is concluded that ultrasonic TWTP technology can achieve welds evidently. The interdiffusion analysis of the molecular motion is studied carefully, which explains the reason for the successes of weld for dissimilar materials.

Composite materials, particularly carbon fiber reinforced polymers (CFRP), have been increasingly used for applications in aircraft structures due to their great potential for weight reduction [152]. In the future, the welding work will be focused on the weld between the composites of CFRC, CNT/polymer composite, and other conductive composites. Also, degradable and medical composites will be considered in future studies due to their needs for the following application.

## Figures and Tables

**Table 1:** Advantages and disadvantages of mechanical fastening.

Advantages	Disadvantages
Reopen ability of WPs	Augmented stress concentration
Convenient technology and machinery	Loosening of WPs due to creep or stress relaxation
Controllable volume capability	Crazing and cracking
Joint of dissimilar materials	Enclosure limitation
Assurance of structural integrity by well-known prediction methods and analysis	residual stresses due to the differences between WPs
Ease of joint inspection	Loss of properties due to moisture
Little surface preparation and cleaning is required	Need to access both sides of the part, increased number of process steps
Repair or replacement of pieces is facilitated	Weight penalty due to thicker sections and fasteners

**Table 2:** Advantages and disadvantages of adhesive bonding.

Advantages	Disadvantages
Dissimilar materials bonding ability	Difficulty to disassemble
Good as repair method	Requiring good surface preparation
No holes required	Assembly rate limitations
Assembly of thin or flexible substrates	Resistant only to shear loading
Low stress concentration	High buying and disposal costs
Good surface finishing	Temperature sensitivity
Weight reduction	Adhesive may suffer thermal and environmental degradation
Sealing	Difficulty in predicting bond failure
Improvement of fatigue resistance	Emission control

**Table 3:** Advantages and disadvantages of USW.

Advantages	Disadvantages
High production rates	Sample size limitations (maximum of 0.23 m × 0.3 m)
Energy efficiency	Usually only for compatible thermoplastics
Design freedom	Noise concerns
Ease of assembly	Expensive equipment
Embedding of extra pieces	
No filler materials needed	
